# Medical Ethics

**Published:** 1851-07

**Authors:** 


### ARTICLE VII.

### *Medical Ethics.*

We extract the following eloquent and just observations relative to the intellectual and moral qualifications which are essential to the worthy exercise of medicine as an art, to the proper social position of the medical profession, and to the maintenance of desirable relations among its members, from an interesting editorial article in the Medical Times. (Dec. 21st, 1850.) *Ed. Med. News and Lib'y, Phila.*

"First, then, we affirm that a high personal character, an elevated tone of feeling, and careful moral culture, are absolutely requisite to form a worthy practitioner of medicine. There is no profession which is so much calculated to try our moral qualities, by bringing us into complex and difficult moral relations with others. The patience and temper of the medical practitioner are continually tried by the fretfulness of illness, by the monotony of iterated complaint, by the garrulity of age, by the childishness of imbecility, by the impertinences of the vulgar, the curiosity of the idle, the prejudices of the ignorant. His equanimity is invaded by the anxieties, the misgivings, the despondency, the agitation, of those who suffer, and of those who are interested in them. His presence of mind and self-dependence are severely tested by sudden dangers, and startling emergencies, and fearful perturbations: by the casualty which crushes manhood in its prime of vigor and usefulness; the attempted suicide; the mental tempest, and moral wreck of madness. His fortitude and his confidence in his own art are sternly taxed by the frequent frustration of his best and wisest efforts. His cheerfulness and tranquillity of mind are overcast by the clouds of sorrow, by the dull night of hopelessness, and the shadow of the tomb settling on all around him. Amid all these troubles and contingencies he must stand unshaken, yet sympathising; serene and gentle, yet active, authoritative and energetic; anxious, and often perplexed himself, yet guiding others in their perplexity, and cheering them in their despondency—himself too heavily laden, yet aiding others to sustain their burden.

"The medical practitioner also must be in the highest degree discreet and trustworthy; he is the depository of more secrets even than the priest, and the peace of many families, and thence the welfare of whole communities, may be entrusted to his keeping.

"Is a man of a narrow heart, of a groveling mind, fit for such ministrations, or worthy of such trust? Surely not. We have here need of one whose being is lofty and spiritualized—purified from the dross of the world—conscious of his high mission, and of his own capacity to fulfil it.

"If there be any truth in these views, it follows that a most essential part of medical education consists in preliminary moral culture; and that, in fact, the very first inquiry to be made in reference to the candidate for medical honors or medical licenses, is, not whether he have studied classics or philosophy—not whether he understand anatomy or chemistry—not whether he have heard lectures or walked hospitals; but whether he can produce unquestionable testimonials of high personal character, and proofs of fine moral culture—whether, in a word, there be good reason to believe that he is individually fit for so serious and so responsible a calling as that of medicine."

Dr. C. A. Harris—Dear Sir—I have extracted the foregoing paragraphs upon medical ethics from an editorial in the Medical Times, republished in the Medical News and Library, and request their admission into your pages, not only on account of the rare excellence of the sentiments which they contain, but also, and especially, for the reason that they are as much needed, and as applicable in all essential respects to the profession of dentistry as to that of general medicine. The dentist, in proportion to his attainments in his science, becomes, of necessity, the medical adviser of his patients. Where he possesses the necessary surgical knowledge, his nice practice and long observation in all the numerous affections of the mouth, teeth, and their dependencies, qualify him for, and his legitimate practice involves him in, the general treatment of his cases both constitutional and local. Indeed, as the study and practice of the healing art now stands divided among its various classes of practitioners, it often happens that the physician and surgeon must call upon the dentist for his aid and direction, while his dependence upon them can scarcely happen, except for the reason of his own professional incompetency. Medical men do not study, nor would it be easy for them to acquire our branch of medical science, but, without a liberal acquaintance with their studies, *we* can practice it only in its lowest and most imperfect offices. The same is true of the oculist and the obstetrician, when their respective sciences are separated, as dentistry from its nature always must be, into distinct specialties of practice. It is because dentistry thus extends into the general domain of medicine for all its principles, and for no inconsiderable share of its practice, that its general institutes and ethics must be regarded as substantially the same. But, my present object is, first, to call attention to the just and beautiful sentiments of the extract; and next, to suggest, for the reflection of your readers, another aspect in which purity and integrity of moral principles should be regarded as indispensable qualifications of medical character. I allude here, not to the direct service which moral excellence renders in the discharge of duty, or to the immediate influence of conscience upon conduct, but to that more subtle, though not less certain action, either for good or ill, of the habitudes of the heart upon the functions of the head. I refer to the tone and temper of mind, to the strength and accuracy of perception and judgment, which the intellect borrows from a thoroughly single honest and earnest soul; and to all that it suffers and loses through the absence of high and pure principles of life. Classification separates mind and feeling for speculative purposes, on good logical grounds; but they are never separated in act and fact without mutual and equal injury. The passions and emotions are the springs of inspiration and life to the gifted mind; and genius takes its power and quality from the heart. The difference between the artist and mechanic may be in *favor* of the duller clay in matters of sheer intellect, but the diviner powers commissioned to serve the world in its highest needs—all that makes the grandest difference between men, is just the difference in their truth and purity of their affections. Other distinctions are not denied, but however considerable, they are only secondary, the distinctions which grow out of the heart, these are primary and absolute. The affections are, in their very nature, the springs of action to the intellect; and it derives all its energy and efficiency from their incitement. If the sensibilities are dull and the feelings sluggish, thought grows sluggish also, and the whole action of the understanding turns heavily upon its wheels. If the heart is selfish, narrow or corrupt, every faculty of the mind feels it like a malady, and betrays it in its perversion; but a pure heart, ennobled by high aims, illuminates and energises with all the power of a natural religion, its associated intellect. Such is the simple philosophy of our nature, and consciousness gives instant and direct testimony of its truth. Thought strongly moved, aspirations nobly aimed, may receive light and knowledge as an influx from without, but the force that animates them is felt to be an innate force. The feelings serve as a medium of reception to high thought, and they refract and distort, or concentrate and intensify the intellectual rays according to their *own* state and structure.

The poet's muse is something more than a figure or memory of an old-time superstition; it is the expression of a conviction, and answers to a fact in his consciousness; the religionist's rapture of emotion opens all the supernatural to his imagination; the artist finds the infinite in the depth of his own sentiments, and all men in their several degrees, feel the elevation which the moral impulses and the inmost instincts of the heart are capable of affording so richly to the action of the understanding. Especially is the truth of this idea obvious in the application I would make of it to the duties of medical life. It is here, if any where, that an honest heart can render service to a tasked mind, and it is here eminently, that the understanding demands consecration from high principles, both for defence and for support. The selfishness, indolence, bigotry and heartlessness that could be better allowed any where else, because less injurious in any other sphere of duty, in medical practice finds easy access and safe shelter. Ignorance and recklessness have greater exemption and more temptation, and the devotion of the best qualities and acutest powers are less conspicuous than in any other service. There is less competency in the criticism, less adequate appreciation of the real merit of a medical man than of any other profession. He may work miracles of skill in the very spirit of an apostle, and those who love him best may know but little of the facts. The comparative feebleness of these influences upon him are indeed replaced by such ennobling confidences, such generous trust, and supporting love and faith in the subjects of his ministry, as have power to fence him round with something more than priestly sanctities; and the hospitalities of heart and home accorded to him are so rich and perfect that they compel an answering fidelity and purity of feeling; but lacking, as his profession does, that sharpness of oversight and observation, that influence of opinion which keeps the intellect of the clergyman, lawyer and politician on the stretch, he needs, more than they all, the prompting, directing and controlling force of strictly moral motives and urgent sentiments of duty to hold him on to the task and urge him on to advancement. Mere mediocrity, ordinary competency and routine punctuality, will keep him safe from the censure of his patient and the public; and his mind needs, therefore, all the more, the truest and severest self-direction and self-government. Moral purity in the conduct of his social life is almost insured to him as a necessary grace of his profession; but no profession needs so much the reflex agency of high integrity of heart upon the mental faculties, and in none are the collateral helps and checks so few and feeble. The mind of the physician is less helped by the discipline of a just criticism from without, and, therefore, needs the more, the higher and purer impulses from within. He needs an enthusiasm, an energy of virtue which shall *well* supply the place of ambition and the discipline of opinion to other men, and endue *him* instead with a principle and a purpose which shall subdue all his selfishness of feeling, support his strength of body, and inspire his faculties of mind.

